# Discriminant Projective Non-Negative Matrix Factorization

**DOI:** 10.1371/journal.pone.0083291

**Published:** 2013-12-20

**Authors:** Naiyang Guan, Xiang Zhang, Zhigang Luo, Dacheng Tao, Xuejun Yang

**Affiliations:** 1 National Laboratory for Parallel and Distributed Processing, School of Computer Science, National University of Defense Technology, Changsha, Hunan, China; 2 Centre for Quantum Computation and Intelligent Systems and the Faculty of Engineering and Information Technology, University of Technology, Sydney, Sydney, New South Wales, Australia; 3 State Key Laboratory of High Performance Computing, National University of Defense Technology, Changsha, Hunan, China; Institute of Psychology, Chinese Academy of Sciences, China

## Abstract

Projective non-negative matrix factorization (PNMF) projects high-dimensional non-negative examples *X* onto a lower-dimensional subspace spanned by a non-negative basis *W* and considers *W^T^ X* as their coefficients, i.e., *X*≈*WW^T^ X*. Since PNMF learns the natural parts-based representation *W*of *X*, it has been widely used in many fields such as pattern recognition and computer vision. However, PNMF does not perform well in classification tasks because it completely ignores the label information of the dataset. This paper proposes a Discriminant PNMF method (DPNMF) to overcome this deficiency. In particular, DPNMF exploits Fisher's criterion to PNMF for utilizing the label information. Similar to PNMF, DPNMF learns a single non-negative basis matrix and needs less computational burden than NMF. In contrast to PNMF, DPNMF maximizes the distance between centers of any two classes of examples meanwhile minimizes the distance between any two examples of the same class in the lower-dimensional subspace and thus has more discriminant power. We develop a multiplicative update rule to solve DPNMF and prove its convergence. Experimental results on four popular face image datasets confirm its effectiveness comparing with the representative NMF and PNMF algorithms.

## Introduction

Dimension reduction uncovers the low-dimensional structures hidden in the high-dimensional data and gets rid of the data redundancy, and thus significantly enhance the performance and reduce the subsequent computational cost. Due to its effectiveness, dimension reduction has been widely used in many areas such as pattern recognition and computer vision. Some data such as image pixels and video frames are non-negative, but conventional dimension reduction approaches like principal component analysis (PCA, [1]) and Fisher's linear discriminant analysis (FLDA, [2]) do not maintain such non-negativity property, and thus lead to a holistic representation which is inconsistent with the intuition of learning parts to form a whole.

Non-negative matrix factorization (NMF, [3]) decomposes a non-negative data matrix *X* into the product of two lower-rank non-negative factor matrices, i.e., *X*≈*WH*. Due to the non-negativity constraints on both factor matrices *W* and *H*, NMF learns parts-based representation and brought much attention in practical tasks such as image processing [4] and data mining [5]–[8]. To utilize the label information of a dataset, Zafeiriou *et al*. [9] proposed Discriminant NMF (DNMF) by incorporating Fisher's criterion to NMF. Guan *et al*. [43] [44] proposed a Nonnegative Patch Alignment Framework (NPAF) that incorporates margin-maximization based discriminative information into NMF. Recently, Guan *et al*. [42] extended NMF to a novel low-rank and sparse matrix decomposition method termed Manhattan NMF (MahNMF). Nevertheless, NMF, DNMF, NPAF, and MahNMF suffer from the out-of-sample deficiency [10] [11], namely it is indirect to obtain the coefficient of any new coming example. Usually, after getting the basis *W* by NMF, we calculate the coefficient of a new coming example *x* as *y* = *W* ^†^ *x*, where *W* ^†^ denotes the pseudo-inverse of *W*. However, such strategy violates the non-negativity property of the coefficients because the pseudo-inverse operator induces negative entries. Conventional dimension reduction methods such as PAF [35], NPE [12] and LPP [13] overcome the out-of-sample deficiency by using the linearization method which learns a projection matrix. They project a new coming example into the lower-dimensional subspace by directly multiplying it with the learned projection matrix.

To overcome the out-of-sample deficiency of NMF, Yuan *et al*. [14] proposed projective NMF (PNMF) based on the linearization method. In particular, PNMF learns non-negative basis of the lower dimensional subspace and considers its transpose as the projection matrix, i.e., *X*≈*WW^T^ X*. Since the learned projection matrix is non-negative, PNMF obtains non-negative coefficient for any new coming example because multiplication of non-negative matrix and non-negative vector produces non-negative vector. In addition, since PNMF implicitly induces *WW^T^*≈*I*, rows of *W* are approximately orthogonal. Moreover, since *W* is non-negative, such orthogonality implies that each column of *W* contains few nonzero entries. Therefore, PNMF implicitly learns parts-based representation. In contrast, NMF never guarantees such parts-based representation [15]. On the other hand, PNMF involves fewer parameters than NMF, and thus it has been widely used in dimension reduction.

Recently, PNMF has been well-studied and extended to deal with various tasks. Liu *et al*. [10] proposed projective non-negative graph embedding (PNGE) which learns two factor matrices, i.e., a non-negative basis matrix and a non-negative projection matrix while PNMF learns a single one. PNGE incorporates both geometric structure and label information in a dataset based on graph embedding [16]. Wen *et al*. [17] proposed orthogonal projective non-negative matrix factorization based on NPE (NPOPNMF) for hyperspectral image feature extraction. However, PNGE and NPOPNMF have two unknown variables like NMF and do not benefit enough from PNMF. To handle non-linear dimension reduction problem, Yang *et al*. [18] proposed non-linear PNMF. Yang *et al*. [18] theoretically analyzed the convergence of the multiplicative update rule (MUR) of PNMF and applied MUR to optimize the non-linear PNMF. Since the objective function of PNMF contains a fourth-order term, MUR suffers from serious non-convergence problem. To remedy this problem, Hu *et al*. [19] approximated PNMF with a high-order Taylor expansion of the objective function and developed a convergent MUR with its convergence proved. To guarantee the convergence of PNMF, Zhang *et al*. [20] solved PNMF by a new adaptive MUR without normalizing the basis matrix in each iteration round.

Although PNMF and its variants have been successfully applied in many fields such as face recognition and document clustering, they share the following problems: PNMF and most of its variants ignore the label information of the dataset, and thus they cannot perform well in classification tasks. PNGE considers the label information based on the graph embedding framework [16], but it induces additional unknown variable and increases the computational complexity. In this paper, we proposed a Discriminant PNMF (DPNMF) to overcome the aforementioned problems. In particular, DPNMF incorporates Fisher's criterion into PNMF to make examples of different classes as far as possible meanwhile make examples of the same class as close as possible in the lower-dimensional subspace. It has been verified that label information enhances recognition performance in practical applications [21]–[24]. Therefore, DPNMF benefits much from the label information and significantly boosts the performance of classification tasks. To avoid the singularity problem in conventional FLDA, DPNMF utilizes a smartly choosing parameter to trade-off both aforementioned objectives. To solve DPNMF, we developed a MUR-based algorithm and proved its convergence. Experimental results on four popular face image datasets including Yale [25], ORL [26], UMIST [27] and FERET [28] confirm the effectiveness of DPNMF comparing with NMF, PNMF and their extensions.

## Analysis

This section surveys both non-negative matrix factorization (NMF) and projective non-negative matrix factorization (PNMF) with their superiorities and shortcomings analysed.

### NMF

Given *n* examples in *m*-dimensional space arranged in a non-negative data matrix 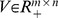, NMF seeks two lower-rank non-negative factor matrices, i.e., 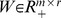 and 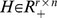, whose product reconstructs *V*. The objective of NMF is to minimize the Kullback-Leiblur (KL) divergence between *V* and *WH*, i.e., 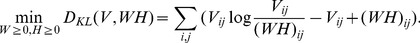
(1)where log signifies the natural logarithmic function. Although NMF is jointly non-convex with respect to *W*and *H*, it is convex with respect to *W* and *H* separately. Therefore, NMF can be solved by alternatively updating both factor matrices. Lee and Seung [3] proposed an efficient multiplicative update rule (MUR) to solve NMF:



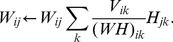
(2)

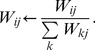
(3)

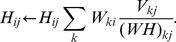
(4)where (2) updates *W* followed by a normalization (3), and (4) updates *H*.

Since NMF ignores the label information of a dataset, it does not perform well in classification tasks. In addition, NMF suffers from the out-of-sample problem because it is non-trivial to calculate the non-negative coefficient of a new coming example.

### PNMF

To overcome the out-of-sample deficiency of NMF, PNMF [14] learns a non-negative projection matrix to directly project *V* onto the lower-dimensional subspace. Let *W* denote the basis matrix, then PNMF treats *W^T^V* as the coefficients and utilize *WW^T^V* to reconstruct *V*. The objective function of PNMF is 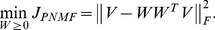
(5)where 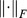 denotes the Frobenius norm. Since *J_PNMF_* is non-convex [19], it is non-trivial to get the global minimum of PNMF. Yuan *et al*. [14] developed a multiplicative update rule (MUR) to iteratively update *W* by
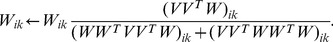
(6)until JPNMF does not change. In each iteration round, PNMF normalizes W by dividing its spectral norm, i.e., 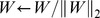 and 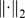 signifies the spectral norm of a matrix, for the following reason. According to (5), PNMF implicitly induces the constraint WWT≈I, which is not guaranteed by (6). The normalization operator shrinks W to make WWT close to I in terms of spectral norm.

PNMF overcomes the out-of-sample deficiency of NMF and learns parts-based representation because it implicitly induces the orthogonality of the learned basis. However, since PNMF ignores the label information of a dataset, like NMF, PNMF does not work well in classification tasks.

## Results

### Discriminant PNMF

**Figure 1:**
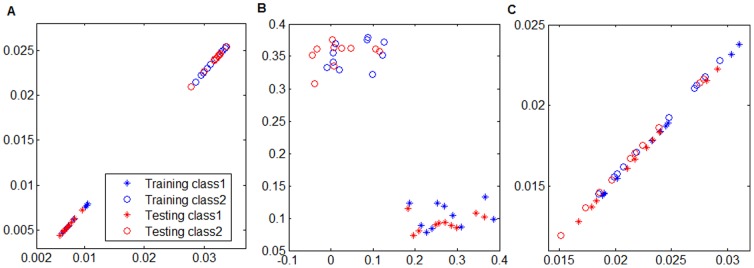
Projected test examples in the learned 2-D subspace. Projected test examples in the learned 2-D subspace by (A) DPNMF, (B) DNMF, and (C) PNMF on the synthetic dataset.

Above analysis gives us two observations on NMF and its extensions: 1) both NMF and DNMF suffer from the out-of-sample deficiency, and 2) although PNMF overcomes the out-of-sample deficiency, it does not utilize the label information in a dataset. To further understand these observations, we sampled 10 training examples and 10 test examples from two 3-D uniform distributions whose means are [0.0137, 0.1009, 0.5292] and [0.0424, 0.2627, 0.326], respectively. We marked both classes of examples by “*” and “o” and obtained totally 20 training examples painted in red and 20 test examples painted in blue in Figure 1. Figure 1.B and Figure 1.C give the projected test examples onto the 2-D subspaces learned by DNMF and PNMF, respectively. Figure 1.B shows that these coefficients contain negative entries caused by the pseudo-inverse operator over the basis matrix, i.e., DNMF suffers from out-of-sample deficiency which weakens its discriminant power. Figure 1.C shows that PNMF overcomes the out-of-sample deficiency but it has weak discriminant power because it completely ignores the label information.

These observations motivate us to take advantages of both DNMF and PNMF and propose Discriminant PNMF (DPNMF) algorithm. In particular, we assume that examples can be projected onto a lower-dimensional subspace and the transpose of basis is considered as a projection matrix. Such assumption implicitly induces parts-based representation of the training examples and overcomes the out-of-sample deficiency like PNMF. To utilize the label information of a dataset like DNMF, DPNMF incorporate Fisher's criteria to enhance the discriminant ability of PNMF. Given training data examples arranged in 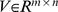, DPNMF learns the basis matrix 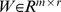
(*r*≤*m* and *r*≤*n*) and projects *V* from *R^m^* to *R^r^* by *W^T^*, i.e., the coefficients *Y* = *W^T^V*. According to [2], DPNMF expects the examples of same class as close as possible and the examples of different class as far as possible in the lower-dimensional subspace. Since *Y* = *W^T^V*, the above two objectives are equivalent to 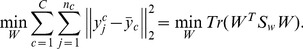
(7)

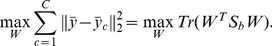
(8)where *C* signifies the number of classes, *n_c_* is the number of examples of class *c*, and 
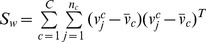
 and 
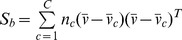
 signify the within-class scatter and between-class scatter, respectively, where 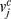 is the *j*-example of class *c*, 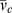 is the mean of examples of class *c*, 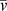 is the mean of all examples. By combining (5), (7), and (8), the objective function of DPNMF is
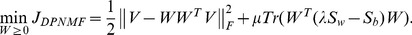
(9)where λ balances objectives (7) and (8), and μ controls the weight of Fisher's criterion.

The tradeoff parameter*λ* is critical in DPNMF (9). According to [29], we choose *λ* as the largest eigenvalue of 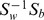, i.e., 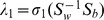, to guarantee the convexity of Fisher's criterion. Although the second term of (9) is convex, the objective function of (9) is non-convex because the loss function of PNMF is non-convex. The following section will present an efficient algorithm to find its local minimum. Another tradeoff parameter *μ* is tuned in the experiments.

### MUR for DPNMF

Since the objective function *J_DPNMF_*(*W*) is non-convex, it is impossible to find its global minimum. Fortunately, it is differential with respect to *W*, and thus the gradient descent method can be used to find a local minimum of (9). By simple algebra, eq. (9) can be written as 
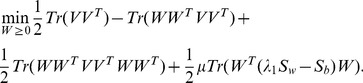
(10)which is obviously a constrained minimization problem. The problem (10) can be solved by using the Lagrangian multiplier method [30]. The Lagrangian function of the objective function of (10) is
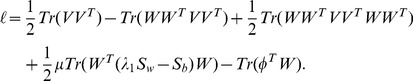
(11)where φ is the Lagrangian multiplier of the constraint W≥0.

According to the K.K.T. conditions [31], the minimizer of (9) satisfies 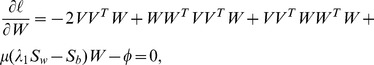
(12)

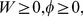
(13)

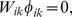
(14)where *W_ik_* stands for the entry positioned at the *i*-th row and *k*-th column of *W*.

By substituting (12) into (14), we have 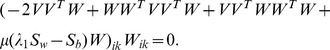
(15)


Since any real matrix *A* can be calculated by its positive items minus the negative items, i.e. 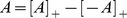, where the operator [*X*]_+_ keeps the non-negative entries of *X* meanwhile shrinks the negative entries to zero, 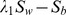 equals to 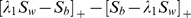 and eq. (15) equals to 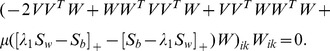



By simple algebra, the above equation is equivalent to 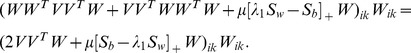
(16)


Eq. (16) gives us a multiplicative update rule (MUR) for DPNMF 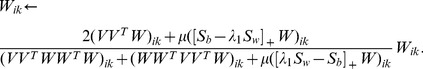
(17)


Since MUR includes only product operators of non-negative matrices, the obtained minimizer naturally satisfies (17). Although MUR is derived from the K.K.T. condition [31], it does decrease the objective function *J_DPNMF_*(*W*) of DPNMF. The following **Theorem 1** proves the convergence of MUR.

**Theorem 1**: The objective function *J_DPNMF_*(*W*) is non-increasing under (17).

We leave the proof of **Theorem 1** in **Materials**.

Similar to PNMF, DPNMF also implicitly induces the constraint *WW^T^*≈*I* which cannot be satisfied by MUR. Therefore, DPNMF normalizes *W* by dividing by its spectral norm in each iteration round to remedy this deficiency. The DPNMF algorithm is summarized in **Algorithm 1** (see Table 1), where the operator 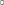 in line 5 signifies element-wise multiplication. The **Algorithm 1** is stopped when the following condition is satisfied: 
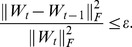
(18)where *t* is the iteration counter and *ε* is a predefined tolerance.

**Table 1:** Summary of MUR algorithm for DPNMF.

Algorithm 1. MUR algorithm for DPNMF
**Input**: Examples 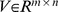 , labels 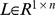 , reduced dimensionality *r*, regularization parameter *μ*.
**Output**: Basis matrix *W*.
1. Calculate *S_w_*and *S_b_* with *V* and *L*, according to (1) and (2), respectively.
2. Calculate the largest eigenvalue *λ*_1_of 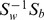 .
3. Initialize 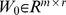 and set *t* = 0.
4. **Repeat**
5. Calculate 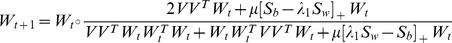 .
6. Normalize 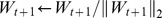 and update 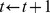 .
7. **Until** {Stopping criterion (18) is satisfied.}.
8. 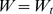 .

The main time cost of **Algorithm 1** is spent on lines 1, 2, and 5. Line 1 constructs both within-class and between-class scatter matrices in *O*(*m* ^2^ *n*) time. Line 2 calculates inverse of *S_w_* and its multiplication with *S_b_* in *O*(*m* ^3^) time. Line 5 denominates the time complexity because it includes multiplications between high-dimensional matrices and the number of iterations is usually large. Looking carefully at line 5, its time costs can be decreased by updating *W_t_*_+1_ by the following two steps: 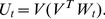
(19)and 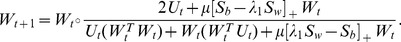
(20)where (19) costs *O*(*mnr*) time and (20) costs *O*(*mr* ^2^+*m* ^2^ *r*) time. Since (20) calculates the shared *U_t_* three times, it saves the time cost of line 5. In summary, the total time complexity of **Algorithm 1** is 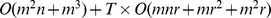, where *T* is the number of iterations, and its memory complexity is 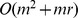.

## Experiments

This section evaluates DPNMF by a comprehensive study of its ability of data representation and its effectiveness in face recognition on four datasets including Yale [25], ORL [26], UMIST [27] and FERET [28] dataset.

### A Comprehensive Study

**Figure 2:**
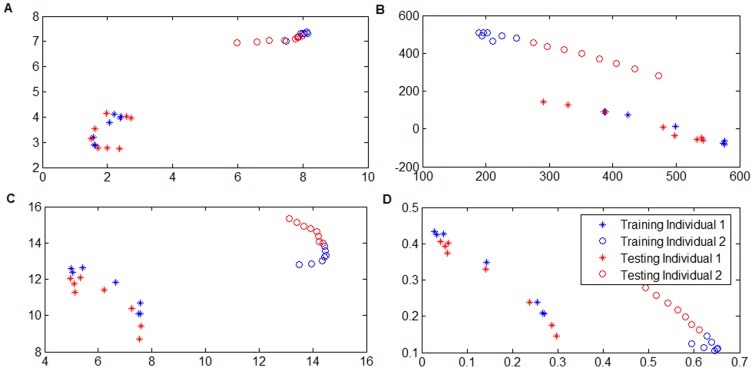
Projected test examples in the learned 2-D subspace on the UMIST dataset. Projected test examples in the learned 2-D subspace: (A) DPNMF, (B) DNMF, (C) PNMF and (D) NMF on the real dataset.

To validate the data representation ability of DPNMF, we conducted a simple experiment before practical tasks. We randomly selected two individuals from UMIST dataset. For each individual, totally 15 images were chosen for this study and 7 images were utilized for training and the remaining 8 images were utilized for testing. Each image was cropped to a 40×40 pixel array and reshaped to 1600-dimensional vector. We marked images of both individuals by “*” and “o”, respectively, and the training images and the test images are painted in blue and red, respectively. Therefore, we obtained totally 14 training images painted in red and 16 test images painted in blue in Figure 2. In this experiment, DPNMF, DNMF, PNMF and NMF were conducted on the training images to learn a 2-dimensional subspace. Then, the test images were projected onto the learned subspace to depict their data representation abilities.

Figure 2 shows the coefficients of both training and test images in the learned subspaces by DPNMF, DNMF, PNMF and NMF. Figure 2.B shows that their coefficients in the DNMF subspace contain negative entries. It means that DNMF suffers from the out-of-sample deficiency, namely the coefficients of the test examples contain negative entries. Figure 2.C shows that PNMF overcomes the out-of-sample deficiency but has weak discriminant power because it ignores the label information of the training images. In addition, NMF suffers from the out-of-sample deficiency and ignores the label information of the training images (see Figure 2.D). Figure 2.A shows that DPNMF simultaneously overcomes the aforementioned drawbacks and separates the images of both individuals perfectly.

### Face Recognition

In this section, we validate the effectiveness of DPNMF by comparing the most related methods including NMF, PNMF, PNGE and DNMF on four datasets including Yale [25], ORL [26], UMIST [27] and FERET [28] dataset. For each dataset, all the face images are aligned according to the position eye. Different numbers of images of each subject were randomly selected to construct the training set and the remaining images consist of the test set. In this experiment, we used the nearest neighbor (NN) rule as a classifier and calculated the accuracy as percentage of test face images that are correctly classified. To eliminate the effect of randomness, we repeated such trial 5 times and compared representative algorithms based on the average accuracy. For DNMF, we set *γ* = 10 and *δ* = 0.0001 over the within class scatter term and between class scatter term, respectively. For PNGE, we set the trade-off parameter *μ* = 0.5 and the other parameters according to [10]. For all algorithms, the maximum number of loops is set to 2000 and the tolerance *ε* of stopping criterion is set to 10^−7^.

Given the training set *V_tr_*, both NMF and DNMF learn a basis *W* and the coefficients as 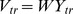. To classify each image *v_ts_*, we first calculate its coefficient 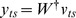 and then classify it to the same class as the image whose coefficient has smallest Euclidean distance to *y_ts_*, i.e., 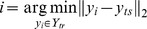. Since both PNMF and DPNMF learn a basis *W* and consider its transpose as a projection matrix, different from NMF and DNMF, the coefficient of a test image *v_ts_* is calculated as 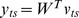. We keep the remaining procedures of classification consistent for fairness of comparison.

**Figure 3:**
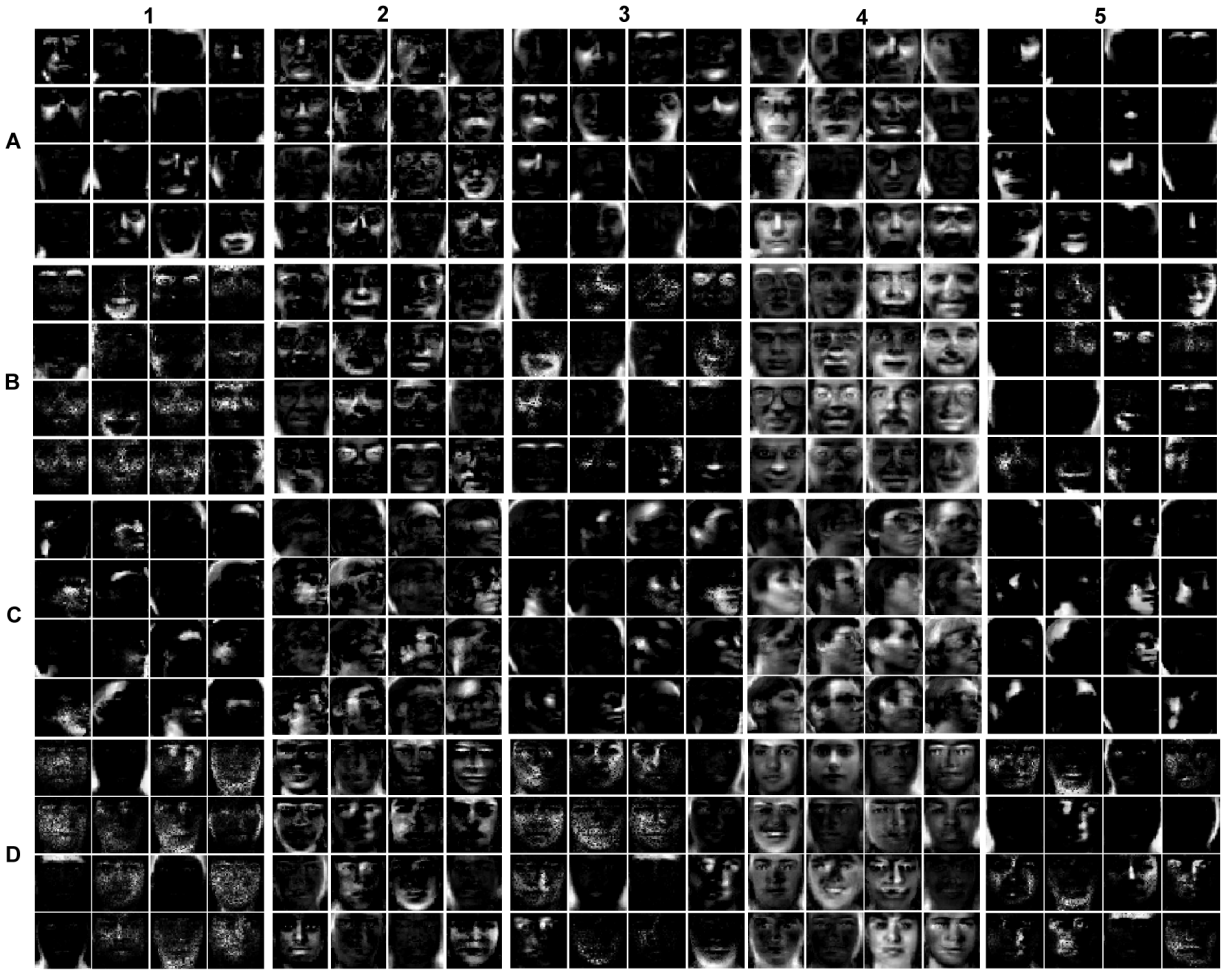
The bases learned by different representative NMF and PNMF algorithms on four popular datasets. The bases learned by (1) DPNMF, (2) DNMF, (3) PNGE, (4) NMF and (5) PNMF on four popular datasets (A) Yale, (B) ORL, (C) UMIST and (D) FERET datasets.

Figure 3 gives the basis images learned by DPNMF, DNMF, PNGE, NMF, and PNMF on Yale, ORL, UMIST, and FERET datasets. It shows that DPNMF learns parts-based representation. In the following, we will validate the effectiveness of such representation.

#### Yale Dataset

**Figure 4:**
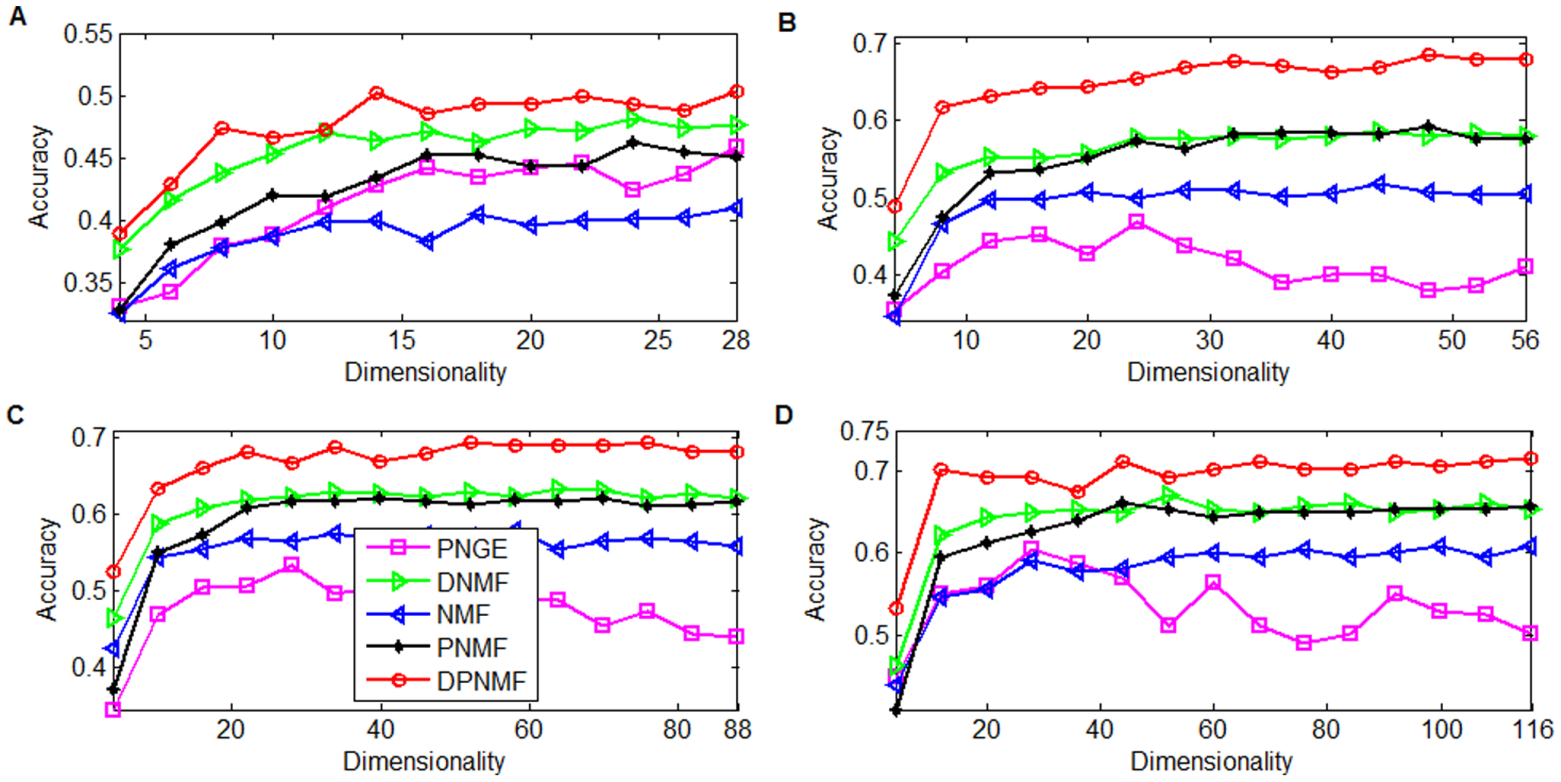
Average accuracies versus different reduced dimensionalities on Yale dataset. Average accuracies versus reduced dimensionalities when (A) 2, (B) 4, (C) 6, and (D) 8 images of each subject of Yale dataset were selected for training.

The Yale face image database [25] consists of 165 grayscale images taken from 15 subjects. Totally eleven images were taken from each subject under different settings such as varying facial expressions (sleepy or surprised) and other configurations. Each image is cropped to 32×32 pixels and reshaped to a 1024-dimensional vector. For each subject, totally 2, 4, 6, and 8 images were randomly selected as the training images and the remaining images as test images. In this experiment, we set the parameter *μ* = 1 for DPNMF (9). Figure 4 reports the average accuracies of DPNMF, DNMF, PNGE, PNMF and NMF on Yale dataset under different settings. It shows that DPNMF significantly outperforms the representative algorithms because it utilizes the label information in representing the training images and such parts-based representation (cf. row A of Figure 3 effectively inhibits the influence of the contained noises.

#### ORL Dataset

The Cambridge ORL database [26] is composed of 400 face images taken from 40 individuals with varying facial expression, lighting and occlusions such as with and without glasses. For each individual, totally 2, 4, 6, and 8 images were randomly selected as the training images and the remaining images as test images. Each image is cropped to 32×32 pixels and reshaped to a 1024-dimensional vector. For DPNMF, the parameter in (9) is set to *μ* = 10 when 2 and 4 images of each individual are selected for training and *μ* = 0.03 when 6 and 8 images of each individual are selected for training.

**Figure 5:**
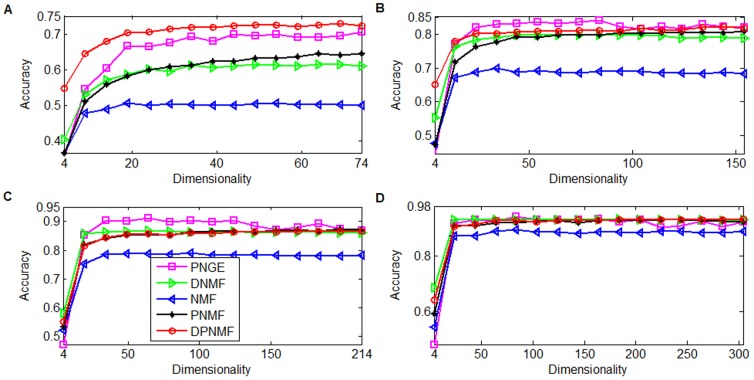
Average accuracies versus different reduced dimensionalities on ORL dataset. Average accuracies versus reduced dimensionalities when (A) 2, (B) 4, (C) 6, and (D) 8 images of each subject of ORL dataset were selected for training.

Figure 5 reports the average accuracies of DPNMF, DNMF, PNGE, PNMF and NMF on ORL dataset under different settings. It shows that DPNMF outperforms DNMF, PNMF and NMF. Figure 5.A shows that DPNMF outperforms PNGE when only two images of each individual are used for training. However, PNGE shows superiority when the training set contains four and six images of each individual (see Figure 5.B and Figure 5.C). That is because the photos in ORL dataset are taken from different views of frontal faces and the local geometric structure enhances the discriminant power of PNGE on such dataset. Figure 5.D shows that DPNMF performs comparably with PNGE when the training set contains eight images of each individual.

#### UMIST Dataset

The UMIST database [27] includes 575 face images collected from 20 individuals from different views and poses. Each image was resized to a 40×40 pixel array and reshaped to a 1600-dimensional long vector. In this experiment, a subset of 300 images composed of 15 images per subject on the left profile was tested. We randomly selected 4, 6, 8, and 10 images from each individual for training and the remaining images are used for testing. For DPNMF, we set the parameter *μ* = 1 in (9) empirically.

**Figure 6:**
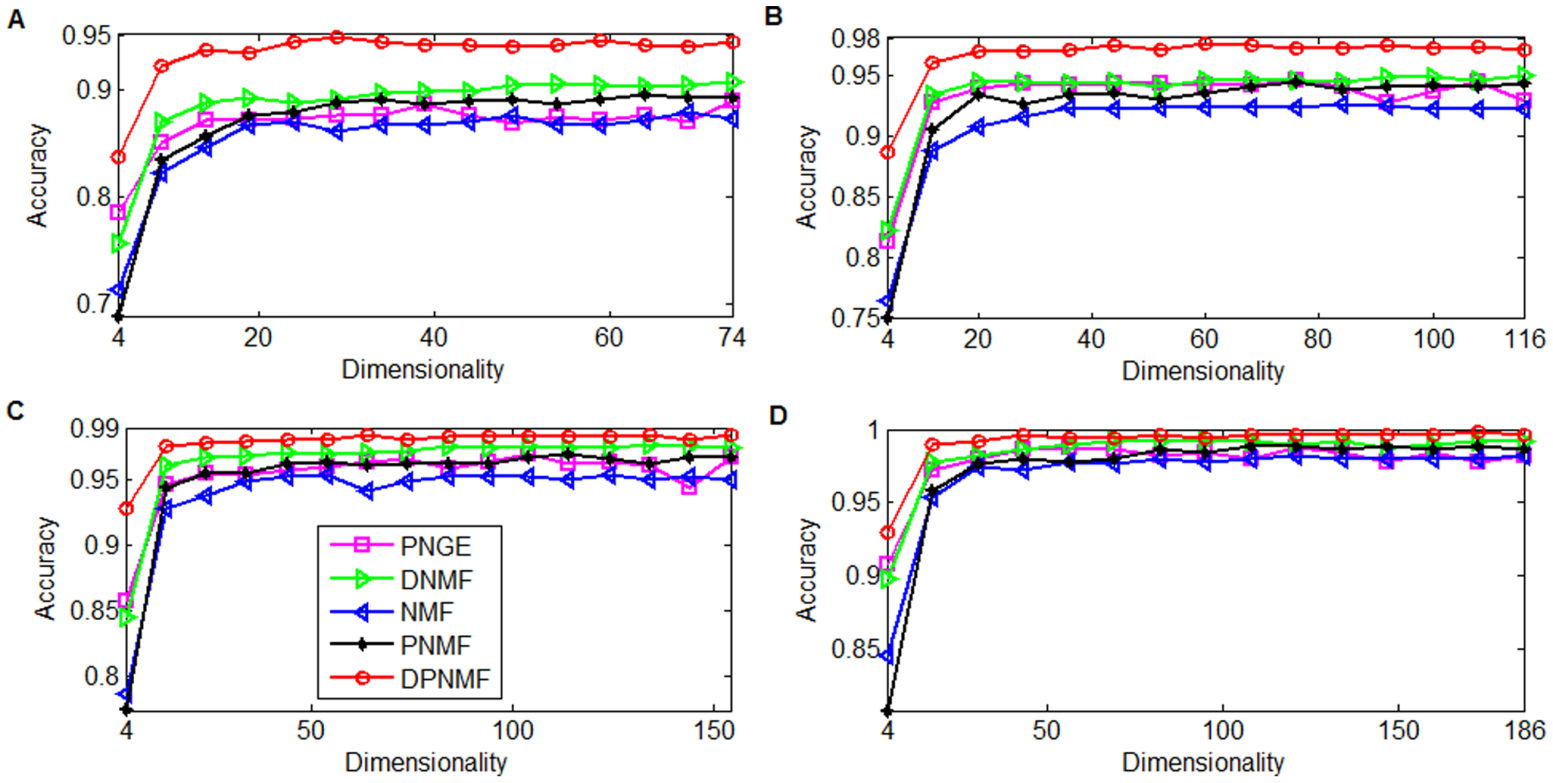
Average accuracies versus different reduced dimensionalities on UMIST dataset. Average accuracies versus reduced dimensionalities when (A) 4, (B) 6, (C) 8, and (D) 10 images of each individuals of UMIST dataset are selected for training.

Figure 6 compares the average accuracies of DPNMF, DNMF, PNGE, PNMF and NMF on UMIST dataset under different settings. It shows that DPNMF significantly outperforms other algorithms especially when four and six images of each individual are selected for training. When eight and ten images of each individual are selected for training, DPNMF almost performs perfectly.

#### FERET Dataset

**Figure 7:**
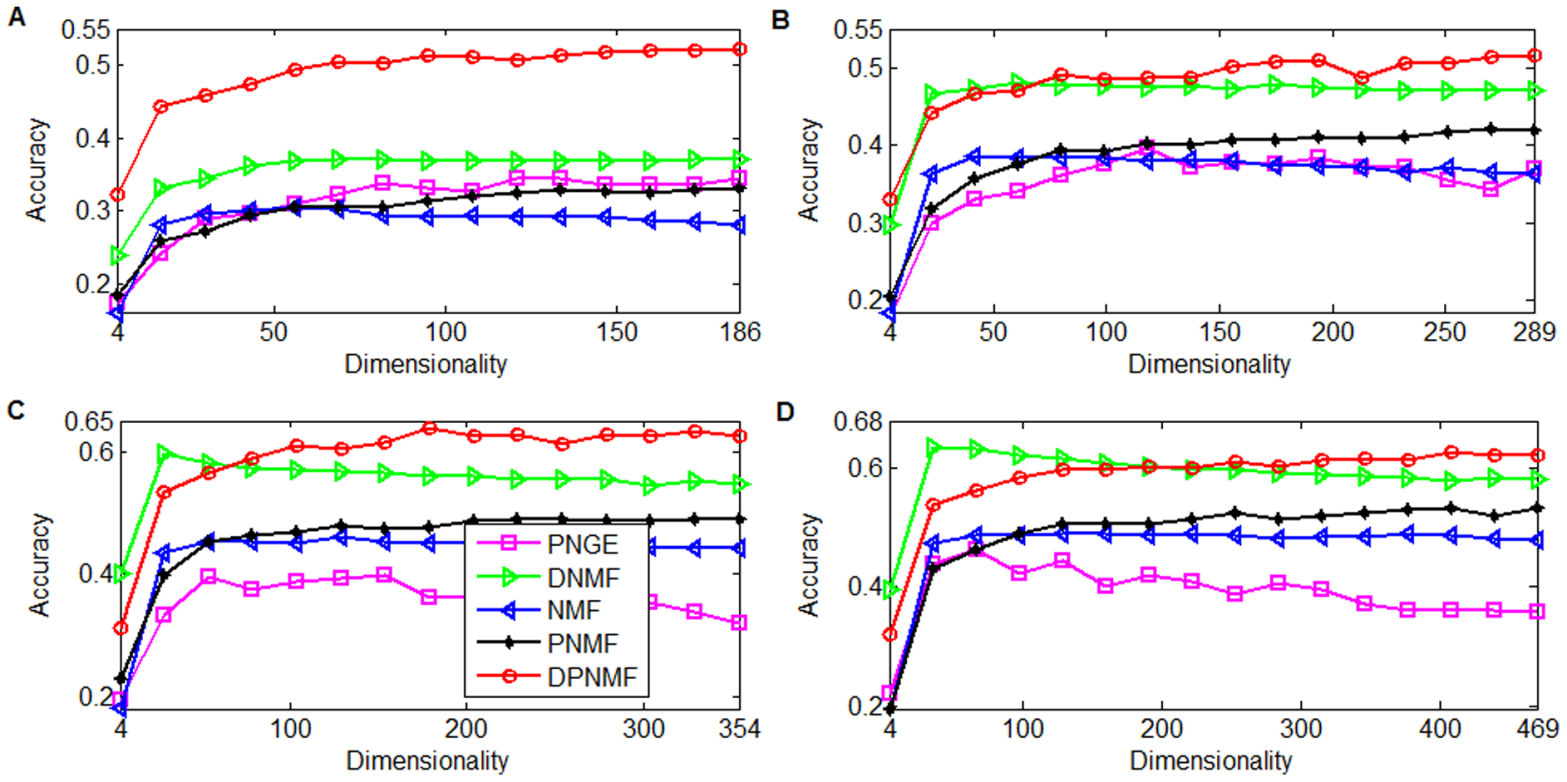
Average accuracies versus different reduced dimensionalities on FERET dataset. Average accuracies versus reduced dimensionalities when (A) 2, (B) 3, (C) 4, and (D) 5 images of each subject of FERET dataset were selected for training.

The FERET database [28] contains 13,539 face images taken from 1,565 subjects varying in size, pose, illumination, facial expression and age. We randomly select 100 individuals and 7 images for each individual to build up the FERET dataset. Each image was cropped to a 40×40 pixel array and reshaped to a 1600-dimensional long vector. Totally 2, 3, 4, and 5 images were randomly selected from each individual for training and the remaining images are used for testing. For DPNMF (9), we set the parameter *μ* = 1 when 2 and 3 images of each individual are selected for training, and set *μ* = 0.1 when 4 and 5 images of each individual are selected for training. Figure 7 reports the average accuracies of DPNMF, DNMF, PNGE, PNMF and NMF on FERET dataset under different settings. It shows that DPNMF significantly outperforms NMF, PNMF, and PNGE because it utilizes the label information in the training set. Figure 7 shows that DNMF also performs well on this dataset especially when 3, 4, and 5 images of each individual are selected for training. However, DNMF performs poorly when only two images of each individual are used for training because the training examples are rather limited in this case and the pseudo-inverse operator over its learned basis greatly reduces the discriminant power of DNMF. DPNMF overcomes such problem, and thus performs well (see Figure 7.A) in this case. Such observation confirms the effectiveness of DPNMF.

## Discussion

This section shows how to tune the tradeoff parameter in DPNMF. In addition, we also give an empirical validation of both convergence and efficiency of the MUR algorithm for DPNMF.

### Parameter Selection

**Figure 8:**
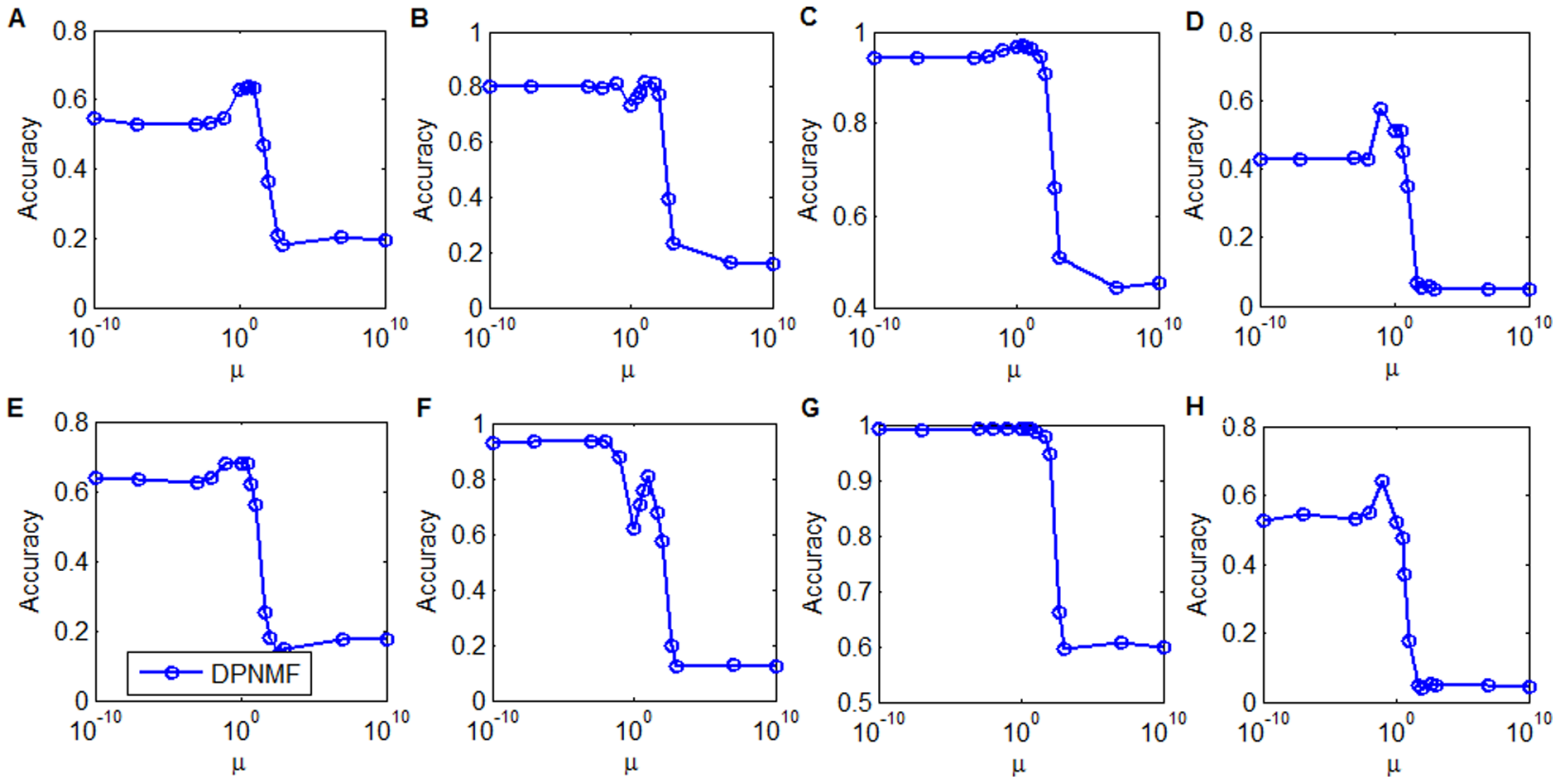
Average accuracies versus the parameter *μ* with the corresponding reduced dimensionality. Average accuracies versus the parameter *μ* when 4 and 8 images of each individual from Yale dataset were selected for training and the reduced dimensionality is set to 50 (A and E), 4 and 8 images of each individual from ORL dataset were selected for training and the reduced dimensionality is set to 120 (B and F), 6 and 10 images of each individual from UMIST dataset were selected for training and the reduced dimensionality is set to 100 (C and G), and 3 and 5 images of each individual from FERET dataset were selected for training and the reduced dimensionality is set to 250 (D and H).

In the proposed DPNMF, there is a trade-off parameter *μ* that controls its discriminant power. It is usually tuned by using grid search on a wide range. In our experiments, we tuned this parameter in a wide range of [10-10 10-7 10-3 0.01 0.1 1 3 5 10 50 100 500 103 107 1010] on the Yale, ORL, UMIST and FERET datasets. To study the consistence of the selected parameter, we randomly select 4 and 8 images from each individual of Yale and ORL datasets for training, and 6 and 10 images from each individual of UMIST dataset for training, and 3 and 5 images from each individual of FERET dataset for training. Such trail is independently conducted five times to eliminate the randomness of training set and the average accuracy is reported in Figure 8.A to Figure 8.H, respectively.

Figure 8.A and Figure 8.E show that DPNMF performs stably when *μ* is selected from 10^−10^ to 1 on the Yale dataset and reaches its peak when *μ* = 1. Figure 7.B and Figure 8.F show that DPNMF performs stably when *μ* varies from 10^−10^ to 0.1 on the ORL dataset and reaches its peak when *μ* = 0.1. Figure 8.C and Figure 8.G show that DPNMF performs stably when *μ* is selected from 10^−10^ to 50 on the UMIST dataset and reaches its peak when *μ* = 3. Figure 8.D and Figure 8.H show that DPNMF performs stably when *μ* is selected from 10^−10^ to 1 on the FERET dataset and reaches its peak when *μ* = 0.01. From Figure 8, we can see that DPNMF performs stably when the parameter *μ* is selected from a wide range, but its discriminant power might decrease when the parameter *μ* is gradually increased. Therefore, we empirically set the parameter *μ* = 1, and this parameter should be tuned for satisfied classification performance on other datasets.

### Convergence Study

**Figure 9:**
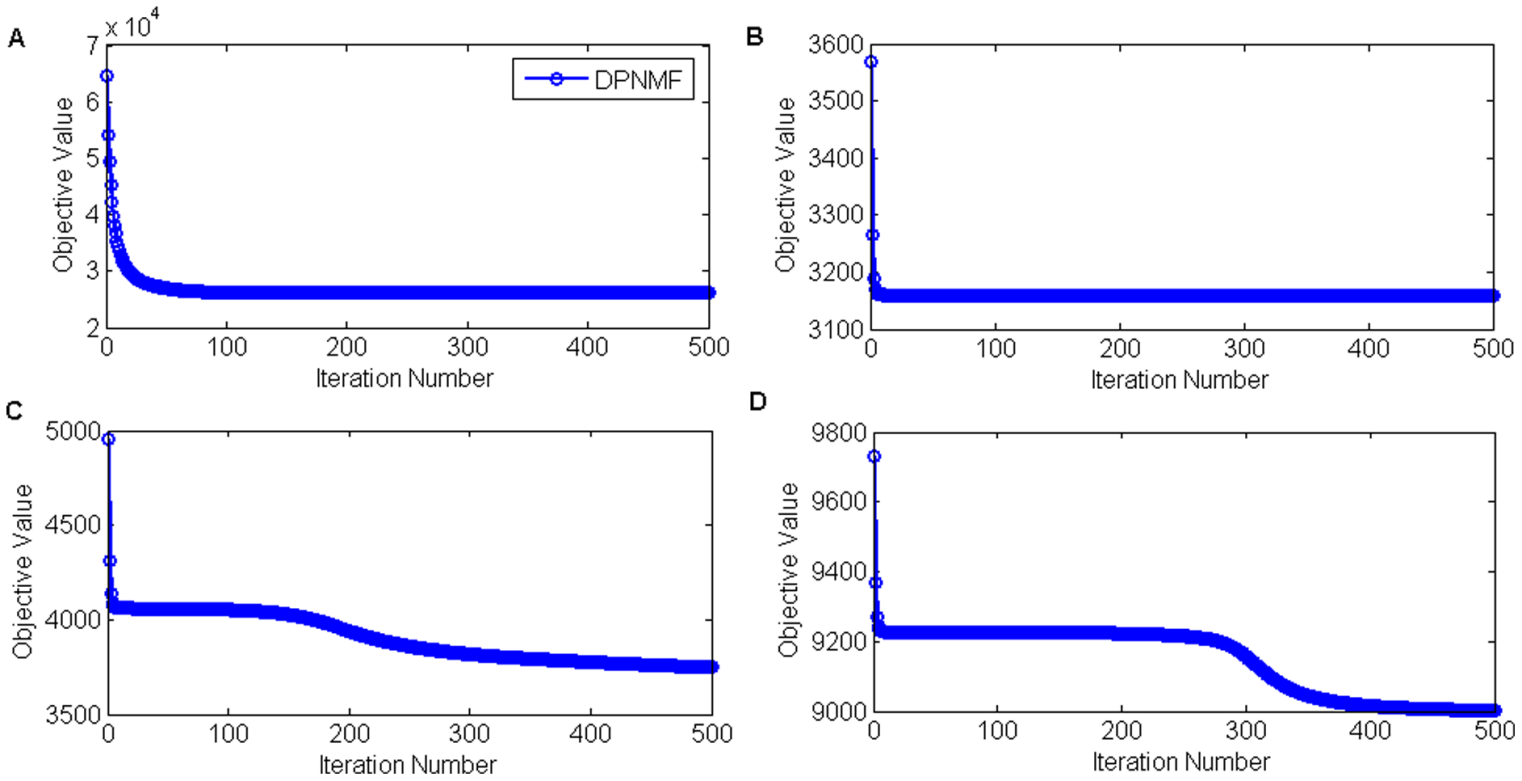
Objective value versus the iterative number on four datasets. Objective value versus the iterative number when (A) 8 images of each individual from Yale datasets, (B) 8 images of each individual from ORL datasets, (C) 10 images of each individual from UMIST datasets, and (D) 5 images of each individual from FERET datasets.

In this section, we verified the convergence of DPNMF on the tested four face datasets. We randomly selected 8, 8, 10 and 5 images from each individual of Yale, ORL, UMIST and FERET datasets for training, and reported the objective values versus numbers of iterations in Figure 9.A to Figure 9.D, respectively. In this experiment, we set the tradeoff parameter *μ* to 10, 0.1, 3, and 0.01, according to above analysis and the reduced dimensionalities to 116, 304, 186, and 496 on the Yale, ORL, UMIST, and FERET datasets, respectively. The maximum number of iterations is set to 500.

From Figure 9.A to Figure 9.D, we can see that MUR gradually reduced the objective function of DPNMF and converges rapidly within 500 iteration rounds on four tested datasets.

### Efficiency Study

**Figure 10:**
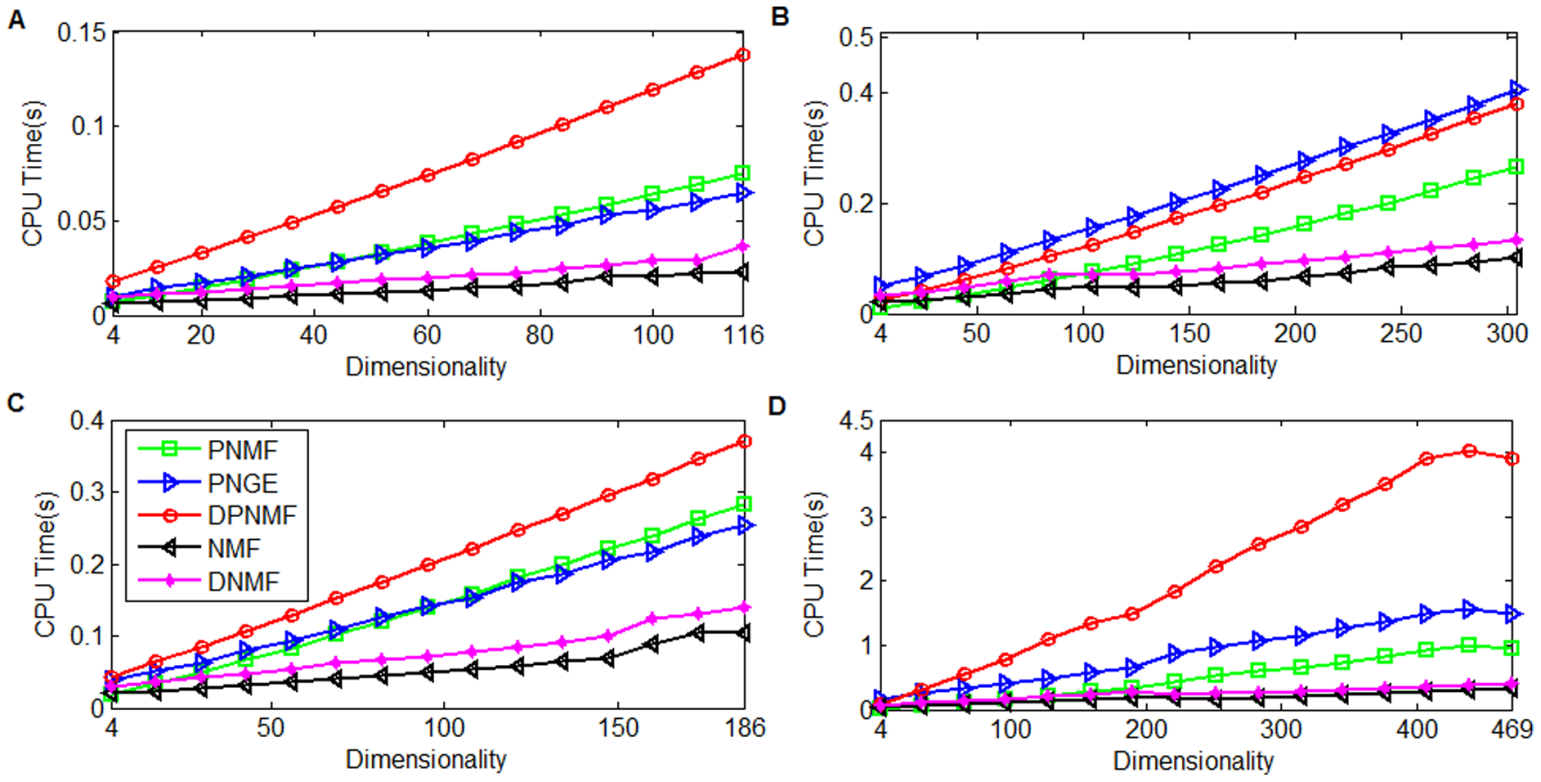
CPU seconds versus reduced dimensionalities on four datasets. CPU seconds versus reduced dimensionalities when (A) 8 images of each individual from Yale datasets, (B) 8 images of each individual from ORL datasets, (C) 10 images of each individual from UMIST datasets, and (D) 5 images of each individual from FERET datasets.

We also verified the computational cost of DPNMF compared with the representative algorithms on Yale, ORL, UMIST, and FERET datasets. Similarly, we randomly selected 8, 8, 10 and 5 images from each individual of Yale, ORL, UMIST and FERET datasets for training and repeated such trial five times to eliminate the effect of randomness. The parameter setting is same as those in above section. We implement all algorithms in MATLAB on a workstation which contains a 3.4 GHz Intel (R) Core (TM) processor and an 8 GB RAM. Figure 10 compares the average CPU costs of each iteration round spent by DPNMF with those spent by PNMF and PNGE on four test datasets.

Figure 10 shows that DPNMF costs more CPU times than the other algorithms because it utilizes two time-consuming operators, i.e., 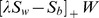 and 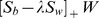 in line 5 of **Algorithm 1**, whose time complexities are both *m* ^2^ *r*. However, DPNMF can achieve higher accuracy than other algorithms (see Figure 4 to Figure 7) due to the incorporated Fisher's criterion. Several excellent NMF optimization algorithms such as NeNMF [45], Online RSA-NMF [46], and L-FGD [47] can be applied to optimize DPNMF more efficiently than MUR.

From above analysis, DPNMF is an effective dimension reduction method. In our future works, we will applied it to many vision tasks, e.g., color to gray image transformation [32], 3-D face reconstruction [33], and 3-D face facial expression analysis [34]. In addition, due to its effectiveness, we will extend DPNMF to tensor analysis [37] for gait recognition [36] and Bayesian model based on covariance learning [38] [39] [40] [41] in our future works.

## Conclusion

This paper proposes an effective Discriminant Projective Non-negative Matrix Factorization (DPNMF) method to overcome the out-of-sample deficiency of NMF and boost its discriminant power by incorporating the label information in a dataset based on Fisher's criterion. We developed a multiplicative update rule to solve DPNMF and proved its convergence. Experimental results on popular face image databases demonstrate that DPNMF outperforms NMF and PNMF as well as their extensions.

## Materials

### Proof of Theorem 1

Given the current solution *W*′, we approximate 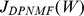 by its Taylor-series expansion 
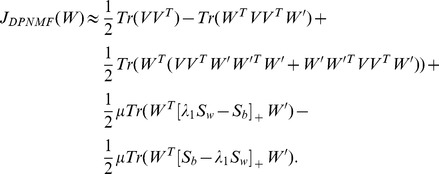
(21)


We construct an auxiliary function 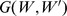 of 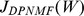 as follows: 
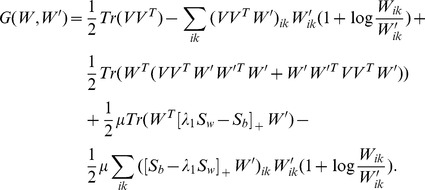
(22)


It is easy to verify that 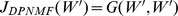.

In the following section, we will prove that 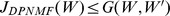 to complete the proof. For any *z*>0, we have 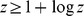. By substituting 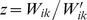 into the above inequality, we have 
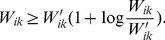
(23)


Since 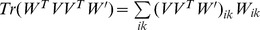 and 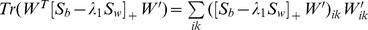, from (23), we have 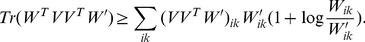
(24)

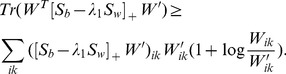
(25)


By substituting (24) and (25) into (21), we prove that 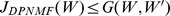.

Assuming *W*″ is the minimum of 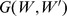, we have the following inequalities: 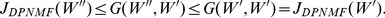
(26)


The remaining things are calculating *W*″ and verifying its non-negativity constraint. To this end, we set the gradient of 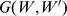 to zero, i.e., 
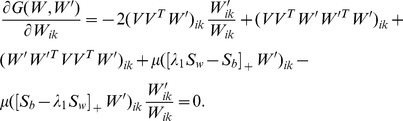
(27)


Eq. (27) gives 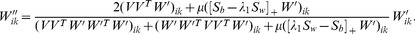
(28)


Since (28) is contains multiplications and divisions of non-negative entries, *W*″ is non-negative matrix.

It is obvious that (28) is equivalent to (17), and thus (26) implies that (17) decreases the objective function of DPNMF. It completes the proof.
